# Cox’s Companion to the Sea Medicine Chest, and Compendium of Domestic Medicine

**Published:** 1851-12

**Authors:** 


*Cox's Companion to the Sea Medicine Chest, and Compendium of Domestic Medicine.* Revised and considerably enlarged. By R. Davis, M. R. C. S. New York: S. S. & W. Wood, 261 Pearl St. 1851. 12mo. pp. 216.

This is a reprint from the *thirty-third* London Edition. It has, thus, for some time, been highly popular in England. It is well adapted for vessels unprovided with a medical officer, and for any persons so situated as to be unable to avail themselves of professional assistance in cases of illness.

